# The Synaptic Extracellular Matrix: Long-Lived, Stable, and Still Remarkably Dynamic

**DOI:** 10.3389/fnsyn.2022.854956

**Published:** 2022-03-08

**Authors:** Tal M. Dankovich, Silvio O. Rizzoli

**Affiliations:** ^1^University Medical Center Göttingen, Institute for Neuro- and Sensory Physiology, Göttingen, Germany; ^2^International Max Planck Research School for Neuroscience, Göttingen, Germany; ^3^Biostructural Imaging of Neurodegeneration (BIN) Center & Multiscale Bioimaging Excellence Center, Göttingen, Germany

**Keywords:** ECM, synapse, plasticity, tenascin, recycling

## Abstract

In the adult brain, synapses are tightly enwrapped by lattices of the extracellular matrix that consist of extremely long-lived molecules. These lattices are deemed to stabilize synapses, restrict the reorganization of their transmission machinery, and prevent them from undergoing structural or morphological changes. At the same time, they are expected to retain some degree of flexibility to permit occasional events of synaptic plasticity. The recent understanding that structural changes to synapses are significantly more frequent than previously assumed (occurring even on a timescale of minutes) has called for a mechanism that allows continual and energy-efficient remodeling of the extracellular matrix (ECM) at synapses. Here, we review recent evidence for such a process based on the constitutive recycling of synaptic ECM molecules. We discuss the key characteristics of this mechanism, focusing on its roles in mediating synaptic transmission and plasticity, and speculate on additional potential functions in neuronal signaling.

## Introduction

An increasing number of studies are showing that synaptic function is strongly influenced by their local environment, including the molecules or cellular components in their vicinity. As a result, the classical synaptic framework (consisting of the pre- and postsynaptic compartments only) has gradually been extended to include the neighboring astrocytic processes (the “tripartite synapse”; Araque et al., 1999) and, ultimately, also the surrounding extracellular matrix (ECM; the “tetrapartite synapse”; Dityatev et al., 2006). Nowadays, the synaptic ECM is recognized to play an essential role in physiological synaptic transmission as well as in plasticity, and its dysregulation has been linked to synaptopathies in a wide variety of brain disorders (Bonneh-Barkay and Wiley, 2009; Pantazopoulos and Berretta, 2016; Ferrer-Ferrer and Dityatev, 2018). An important property of this ECM is that its molecules are among the longest-lived in the brain, which renders this structure extremely stable (Toyama et al., 2013; Dörrbaum et al., 2018; Fornasiero et al., 2018), and while this quality makes the ECM well-suited to provide long-term support to synapses, both functionally and structurally, it is seemingly ill-suited to allow for very frequent synaptic changes. However, increasingly more studies are showing that changes to synaptic structure can be extremely frequent, even in the adult brain (Berning et al., 2012; Willig et al., 2014; Wegner et al., 2018). In light of these observations, one would expect a mechanism to be in place for maintaining sufficient flexibility of the ECM at synapses, to allow for ongoing structural plasticity. In this review, we discuss a novel mechanism proposed to provide such flexibility, in the form of molecular recycling of ECM components at synapses (Dankovich et al., 2021). We begin by briefly reviewing the various roles of ECM components at the tetrapartite synapse and the existing model for ECM remodeling, followed by a discussion on the plausibility of ECM recycling and its potential implications for our current understanding of synaptic signaling.

## Organization of The ECM at Synapses

In the adult brain, the major components of the neuronal ECM are a family of chondroitin sulfate proteoglycans (CSPGs) called lecticans, and their binding partners: the glycoprotein tenascin-R (TNR) and the glycosaminoglycan hyaluronic acid. Together, these organize into an extensive lattice where long chains of hyaluronan form a backbone for lecticans to bind, and these are thoroughly cross-linked through extensive interactions with TNR (Ruoslahti, 1996). Hyaluronan remains attached to the transmembrane synthase that produces it, which effectively tethers these structures to the surface of the plasma membrane (Dityatev et al., 2010; Sorg et al., 2016). ECM lattices can be found throughout neuronal surfaces, albeit with variations in the relative abundance of the various components and the density of these structures. Particularly dense conformations can be found in the form of perineuronal nets (PNNs) that enwrap the soma and proximal dendrites of a subgroup of neurons, while more diffuse conformations are found pan-neuronally, including finer segments of the neurites and the perisynaptic spaces (Dityatev and Schachner, 2003). In addition to secreted molecules, synapses are also associated with a variety of membrane-bound molecules that interact with the nearby ECM. One well-studied example is the integrin family of ECM receptors, which play an important role in the modulation of actin-associated proteins, and therefore act as a link between the ECM and the neuronal cytoskeleton, allowing these ECM ligands to act as modulators of synaptic structure (Shi and Ethell, 2006; Park and Goda, 2016). Besides the various ECM receptors that are present in the synaptic membrane, there is also growing evidence that many membrane-bound components of the synaptic transmission machinery, such as neurotransmitter receptors, can interact with ECM molecules at the synapse. In the following section, we review some of this evidence, and discuss the potential role of these interactions in modulating various aspects of synaptic function.

## Roles of The ECM at The Tetrapartite Synapse

### Stabilization and Maintenance of Synapses

**Figure 1:**
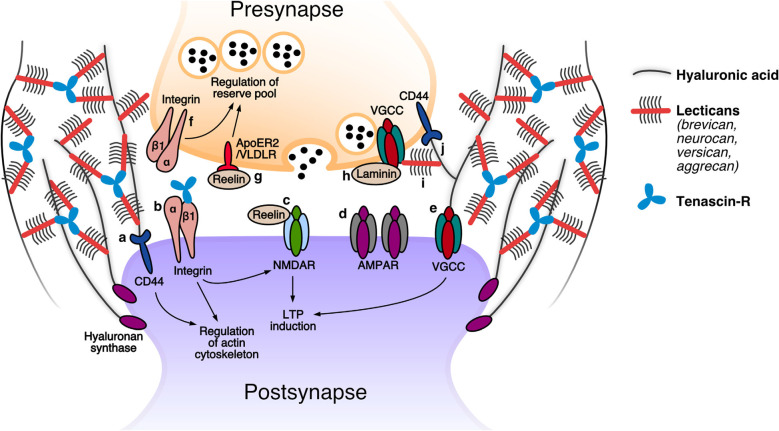
The tetrapartite synapse: speculated roles of extracellular matrix (ECM) molecules. **Postsynapse: (a)** The receptor for hyaluronan CD44 was demonstrated to modulate the activity of actin-associated proteins and thereby promote the stabilization of dendritic spines (Roszkowska et al., 2016). **(b)** β1 integrin receptors are proposed to contribute to LTP by modulating NMDAR-mediated currents, and play a role in spine restructuring and stabilization by modulating the actin cytoskeleton. An interaction has been reported between β1 integrins and tenascin-R (TNR) (Bernard-Trifilo et al., 2005; Liao et al., 2008; Tan et al., 2011; Sloan Warren et al., 2012). **(c)** Increased levels of reelin augment NMDAR-mediated currents, leading to enhanced LTP responses (Weeber et al., 2002; Rogers et al., 2011). **(d)** The hyaluronan-based perisynaptic ECM restricts the lateral mobility of AMPARs (Frischknecht et al., 2009). **(e)** Hyaluronan regulates VGCC-dependent plasticity by modulating these channels (Kochlamazashvili et al., 2010). **Presynapse: (f)** Presynaptic β1 integrin receptors are presumed to regulate the reserve pool of synaptic vesicles (Huang et al., 2006). **(g)** Reelin activation of its ApoER2 and VLDLR receptors modulates reserve pool synaptic vesicles (Bal et al., 2013). **(h)** Presynaptic active zone proteins are anchored at the membrane through putative interactions with β2 laminins (such as with VGCCs; Nishimune et al., 2004; Hunter et al., 2019). **(i)** The presence of perisynaptic brevican is essential for a correct alignment of presynaptic VGCCs in front of the postsynaptic density (Sonntag et al., 2018). **(j)** CD44, the receptor for hyaluronan, was shown to be present presynaptically and is essential for presynapse stability (Roszkowska et al., 2016). Modified from Dankovich (2021).

Expectedly, the perisynaptic ECM provides a steric hindrance to the diffusion of transmembrane molecules at the synapse (Figure 1). *In vitro*, postsynaptic AMPA-type glutamate receptors become significantly less mobile after ~2–3 weeks in culture, which also corresponds to the time at which structured ECM begins to appear on the neuronal surfaces (Borgdorff and Choquet, 2002; John et al., 2006). Disrupting the ECM through enzymatic cleavage of hyaluronan was shown to partially restore this juvenile level of mobility (Frischknecht et al., 2009). Interestingly, this effect was not limited to AMPA receptors, since the authors also reported a reduction in the mobility of green fluorescent protein (GFP) that was artificially introduced into the membrane, suggesting that the ECM at synapses stands as a diffusion barrier to a wide variety of membrane-associated proteins (Frischknecht et al., 2009). For the presynapse, evidence for ECM-mediated stabilization of membrane proteins comes from studies of synapses in the auditory pathway. In cochlear inner hair cell synapses, a deficiency in the lectican brevican leads to a misalignment of presynaptic calcium channels, resulting in a mild hearing loss (Sonntag et al., 2018). In the calyx of Held synapses, a loss of brevican results in altered dynamics in synaptic transmission that are also consistent with a change in the organization of presynaptic calcium channels (Blosa et al., 2015). Taken together, these findings suggest that the ECM-imposed hindrance of diffusion is necessary for the functional organization of synaptic transmission machinery.

In addition to hindering protein diffusion, it also appears that the ECM constricts the mobility and outgrowth of the synapse itself. Application of CSPG-cleaving enzymes *in vitro* and *in vivo* has been shown to result in the outgrowth of dendritic spine heads, and an enhancement of spine motility (Orlando et al., 2012; de Vivo et al., 2013). Mechanistically, such a treatment may act not only to release the constraint placed by the ECM, but is also likely to interfere with direct interactions between ECM molecules and synaptic transmembrane proteins that contribute to synapse stabilization. For example, integrin receptors containing the β1 subunit are known to promote spine maintenance through the modulation of the actin cytoskeleton, and are also known to interact with TNR and CSPGs (Liao et al., 2008; Tan et al., 2011; Sloan Warren et al., 2012). Similarly, the hyaluronan receptor CD44 has been shown to affect spine structure through its modulation of actin cytoskeleton regulators. Furthermore, a knockdown of this receptor was shown to reduce the number of presynapses labeled by the active zone marker bassoon (Roszkowska et al., 2016). It remains to be determined whether the interaction of these ECM receptors with their ligands is necessary for their stabilization of the synapse.

### Modulation of Postsynaptic Plasticity

ECM molecules have also been shown to directly modulate the activity of machinery involved in synaptic plasticity (Figure 1). In many synapses, plasticity-related changes are instigated through the activity-dependent opening of NMDA receptors (NMDARs), which results in an influx of calcium and, subsequently, the long-term potentiation of postsynaptic responses (LTP; Herring and Nicoll, 2016). A number of studies have demonstrated that the activation of postsynaptic β1 integrins is necessary for the initiation and maintenance of LTP, both by modulating the actin cytoskeleton to allow dendritic spine head enlargement and, presumably, resulting in an augmentation of NMDA-mediated currents (Bernard-Trifilo et al., 2005; Kramar et al., 2006; Rex et al., 2009). Accordingly, mice that harbor a neuron-specific deficiency in β1 integrins have impaired NMDAR-dependent LTP (Chan et al., 2006; Huang et al., 2006). It should be noted, however, that the demonstration of an integrin-dependent modulation of NMDARs relied on short integrin ligand (RGD) peptides that have since been shown to directly act on these receptors (Cingolani et al., 2008). It, therefore, remains to be established whether such modulations also take place at the physiological level.

An additional ECM component that has been implicated in LTP is the secreted glycoprotein reelin (generally known for its role in early brain development; D’Arcangelo, 2014). Several studies have shown that reelin supplementation results in enhanced LTP responses, likely due to its ability to modulate NMDAR-mediated currents. In addition, mice deficient in reelin were found to have impairments in LTP (Weeber et al., 2002; Beffert et al., 2005; Qiu et al., 2006; Rogers et al., 2011). Lastly, it is worth mentioning that additional, NMDAR-independent LTP mechanisms have also been linked to ECM modulation. For example, both hyaluronan and tenascin-C were shown to modulate a form of LTP that depends on signaling through postsynaptic L-type voltage-gated calcium channels (LVGCCs; Evers et al., 2002; Kochlamazashvili et al., 2010).

Besides *bona fide* plasticity mechanisms, synapses also have “metaplasticity” mechanisms in place that allow them to modify their predisposition to undergo plasticity. This is often achieved through an adjustment of a neuron’s basal level of excitation, which can act to temper the threshold for LTP induction (Abraham and Bear, 1996). The ECM glycoprotein TNR has been linked to such metaplasticity mechanisms due to its ability to modulate GABA-mediated inhibitory transmission, an important determinant of basal neuronal activity. TNR-deficient mice have elevated levels of basal excitatory transmission and hence a metaplastic increase in the LTP induction threshold (Saghatelyan et al., 2001; Nikonenko et al., 2003; Bukalo et al., 2007). It is possible that TNR exerts its modulation through direct interaction with GABA_B_ receptors (Kruse et al., 1985; Saghatelyan et al., 2001, 2003).

### Modulation of Synaptic Vesicle Release

As for the postsynapse, studies have also demonstrated that the ECM can directly modulate the presynaptic machinery involved in synaptic vesicle release (Figure 1). Recent evidence suggests that laminins, which have largely been studied in the context of brain development, are essential for the organization of presynaptic release machinery at synapses in the adult brain. In the retina, a deficiency in laminin β2 disrupted the spatial organization of a variety of presynaptic components, while their expression level remained unchanged (Hunter et al., 2019). It is possible that laminin β2 molecules achieve this function through direct interactions with the extracellular region of one or more of these components (e.g., they are known to bind presynaptic calcium channels at neuromuscular junction synapses; Nishimune et al., 2004). In addition to prospective interactions with release machinery, laminins may also interact with the synaptic vesicles themselves. At neuromuscular junctions, laminin α5 subunits were found to interact with the synaptic vesicle protein 2 (SV2), which plays a role in priming synaptic vesicles for their release (Son et al., 2000; Chang and Sudhof, 2009). Since laminin α5 was recently also shown to be present at central synapses, it is possible to imagine that it also plays a role in synaptic vesicle release in the brain (Omar et al., 2017). Besides these direct interactions with key synapse components, it is also possible that laminins carry out some of their functions indirectly, through an interaction with ECM receptors such as integrins (Carlson et al., 2010; Nirwane and Yao, 2018). For example, β1 integrins (known to bind laminin α5) were shown to be present at hippocampal presynapses (Mortillo et al., 2012). Furthermore, a neuron-specific deficiency in β1 integrin results in altered synaptic responses that are congruent with a reduced mobilization of vesicles belonging to the reserve pool (i.e., vesicles that are released only rarely under physiological conditions; Huang et al., 2006).

An additional ECM molecule that has been implicated in the modulation of synaptic vesicle release is reelin. A study by Bal and colleagues demonstrated that the application of reelin *in vitro* results in a significant increase in spontaneous vesicle release. Evidence suggests this is due to an increase in presynaptic calcium levels, possibly as a result of the interaction between reelin and its receptors ApoER2 and VLDLR (Bal et al., 2013). Interestingly, the authors also found that reelin specifically mobilizes vesicles enriched with the synaptic vesicle protein VAMP7, which are generally believed to be “reserve pool” vesicles (Hua et al., 2011). Similar to the findings for β1 integrins described above, this demonstrates that the ECM is capable of differentially modulating synaptic vesicle pools.

## ECM Remodeling at The Synapse

Since the ECM is integral to synapse stabilization and maintenance, events of synaptic plasticity are likely to require extensive remodeling of these components at synapses. The currently prevailing notion is that ECM remodeling takes place through proteolytic cleavage of these molecules by locally secreted enzymes, followed by the integration of newly-synthesized ECM molecules. One well-studied example is the local synaptic secretion of matrix metalloproteinase 9 (MMP9) at the onset of LTP, which was shown to be necessary for the accompanying structural plasticity of dendritic spines (Nagy et al., 2006; Wang et al., 2008; Gawlak et al., 2009; Michaluk et al., 2011; Dziembowska et al., 2012). MMP9 can be subsequently deactivated through the parallel secretion of tissue inhibitor of metalloproteinase1 (TIMP1), allowing this cleavage to be transient (Okulski et al., 2007; Magnowska et al., 2016).

**Figure 2:**
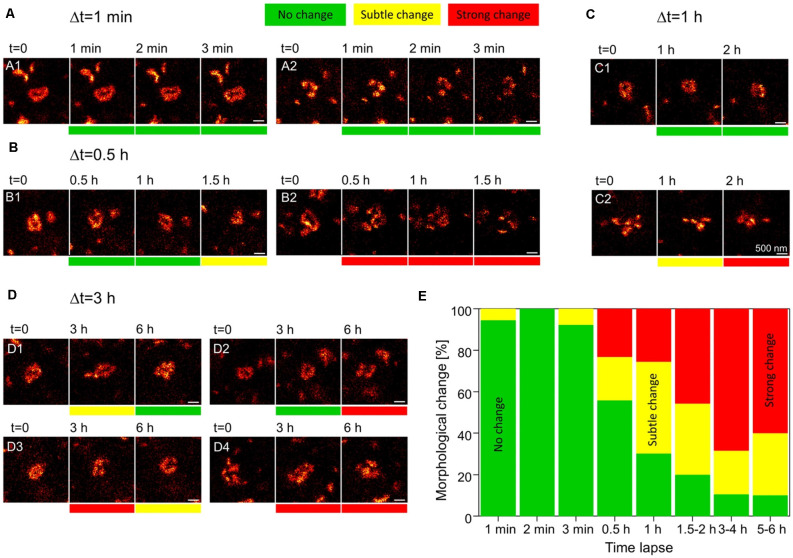
Rapid dynamics of the postsynaptic density (PSD) in dendritic spines. Knock-in mice in which the postsynaptic density protein PSD95 was fused to a fluorescent reporter were used to track the morphology of the PSD. Individual dendritic spines in the visual cortex were imaged using live stimulated emission depletion (STED) microscopy for up to 6 h. **(A)** At a time interval of 1 min, no morphological changes to PSD assemblies are observed. **(B,C)** At higher intervals of 30 min to 2 h, morphological changes can be seen at some synapses **(B2,C2)**, while others appear to remain stable **(B1,C1)**. **(D)** At synapses imaged up to 6 h, PSD assemblies may undergo morphological changes and then return to their original structure **(D1)**, remain unchanged for several hours and only then undergo a morphological change **(D2)**, or undergo multiple morphological changes over the course of several hours **(D3,D4)**. Scale bars = 500 nm. **(E)** Quantification of the % of either no change, subtle and strong changes to the morphology of postsynaptic assemblies of PSD95 for increasing imaging time intervals. *N* = 4 mice; *n* = 18 (1–2 min), 13 (3 min), 43 (0.5–1 h), 35 (1.2–2 h), 10 (5–6 h) PSD95 assemblies imaged. Adapted with permission from Wegner et al. (2018; http://creativecommons.org/licenses/by/4.0/).

While proteolysis-dependent ECM remodeling comprises a tightly controlled mechanism for mediating synaptic plasticity, it is expected to become metabolically expensive when employed very frequently. It is therefore difficult to reconcile this mechanism with the emerging understanding that structural synaptic plasticity is an extremely frequent event: as demonstrated by multiple super-resolution imaging experiments, synaptic morphology can change drastically within just minutes to hours (e.g., Figure 2; Berning et al., 2012; Testa et al., 2012; Willig et al., 2014; Wegner et al., 2018). If every structural fluctuation at synapses were to involve proteolysis and *de novo* synthesis of the ECM, this mechanism would necessitate a relatively fast turnover of these molecules, bringing them close to the lifetimes of other synaptic components that are affected by plasticity, as the postsynaptic receptors. Nevertheless, experimental evidence suggests that ECM molecules are among the longest-lived in the brain (see Table 1 below, for examples of ECM protein lifetimes *in vivo*, Toyama et al., 2013; Dörrbaum et al., 2018; Fornasiero et al., 2018), far longer-lived than the average pre- or postsynaptic protein. It is, therefore, highly likely that additional mechanisms of ECM remodeling exist that do not require a continual turnover of ECM molecules.

**Table 1:** Average lifetimes of select extracellular matrix (ECM) and synaptic proteins (as reported in Fornasiero et al., 2018).

Protein	Half-life in adult mouse brain (days, range for several regions shown)
Brevican	17–31
Neurocan	20–97
Aggrecan	24
Versican	49–687
Tenascin-R	39–74
Synaptic proteins:	
SNAP25	3–4
VAMP2	11–15
Synaptotagmin1	9–10
NMDA receptors	3–7
AMPA receptors	6–15
PSD95	13–16
Homer1	13–14

## Recycling of Synaptic ECM

**Figure 3:**
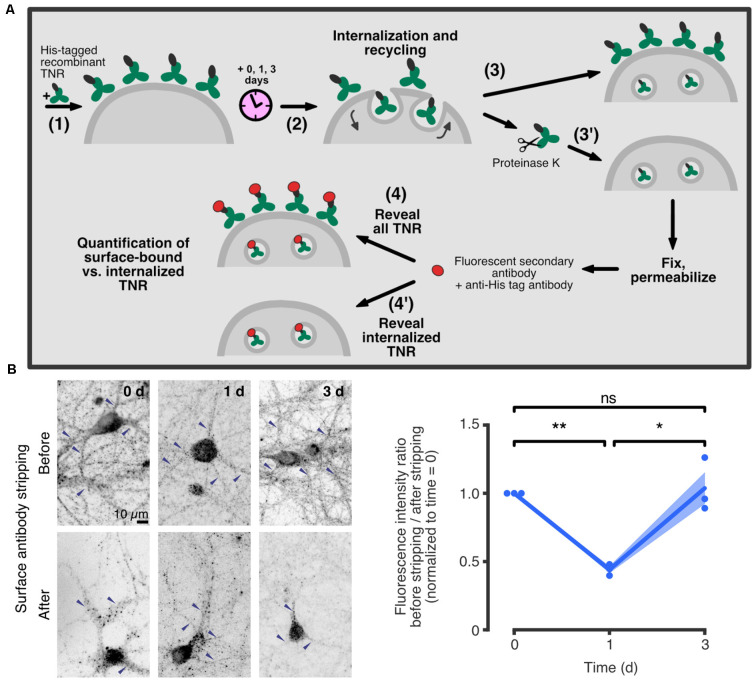
TNR recycles in neurons over ~3 days. **(A)** A schematic of an assay to assess TNR recycling. (1) Cultured hippocampal neurons were pulsed with recombinant His-tagged TNR, which was then allowed to potentially internalize and recycle for a period of 0–3 days (2). After the incubation period, the neurons were immediately fixed (3), or fixed following treatment with proteinase K to strip away all surface-bound recombinant TNR molecules (3’). Following permeabilization treatment, the neurons were immunostained using antibodies against the His-tag to visualize all recombinant TNR (4), or the internalized recombinant TNR only (4’). **(B)** Immediately after pulsing the neurons with recombinant TNR, the staining was visibly reduced by the surface stripping, indicating that the majority of the molecules were surface-bound. One day after the pulse, the signal was similar for non-stripped and stripped neurons, indicating that most molecules had been internalized. Three days after the pulse, surface stripping visibly reduced the staining once again, indicating that a portion of recombinant TNR molecules had recycled back to the surface. Blue arrowheads indicate labeledTNR in neurites. Scale bar = 10 μm. Statistical significance was evaluated with repeated-measures one-way ANOVA (*F*_1.044, 2.088_ = 28, 6, *p = 0.03), followed by Fisher’s LSD (“0 days” vs. “1 day”: **p = 0.002; “1 day” vs. “3 days”: *p = 0.027; “0 days” vs. “3 days”: *p* = 0.775). *N* = 3 independent experiments. In the plot, lines represent the means, shaded areas represent the SEM, and dots represent individual experiments. Adapted from Dankovich et al. (2021) with permission from Springer Nature (http://creativecommons.org/licenses/by/4.0/).

A novel mechanism of ECM remodeling was presented in a recent study by Dankovich and colleagues, based on the recycling of ECM molecules at the synapse. The authors proposed that these molecules are constitutively internalized into neurons, and subsequently resurface and re-integrate into the ECM around synapses (Dankovich et al., 2021). A complete recycling loop was described for the glycoprotein TNR, which spans ~3 days (Figure 3).

**Figure 4:**
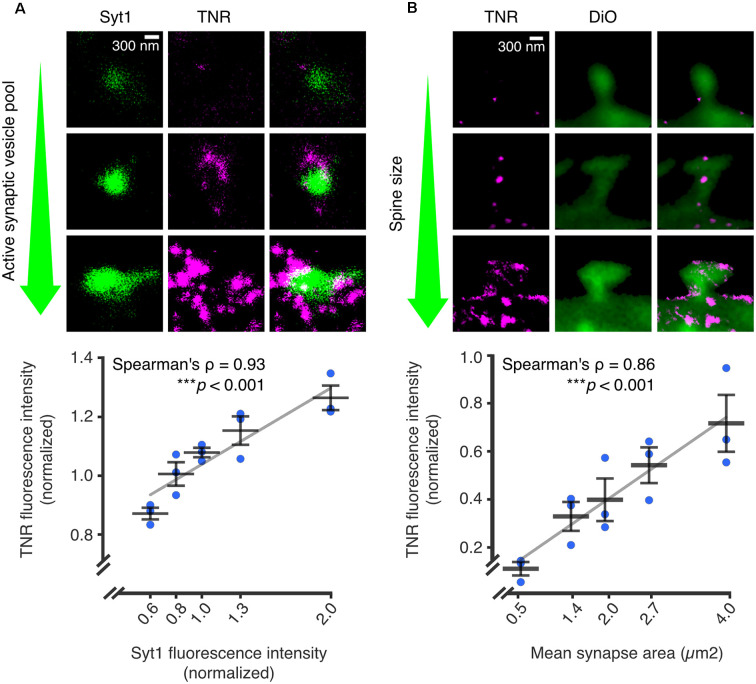
The abundance of recycling TNR molecules at synapses is correlated to synaptic weight. Recycling TNR epitopes were labeled using a live immunostaining-based assay. First, all surface-bound epitopes are blocked with unlabeled antibodies against TNR. After a period of time, newly-emerged epitopes are revealed with the same TNR antibodies conjugated to fluorophores. **(A)** Newly-emerged TNR epitopes were labeled 12 h after surface-blocking (magenta). At the same time, actively recycling synaptic vesicles were labeled with antibodies against the lumenal domain of synaptotagmin1 (Syt1; green), as a proxy for synaptic activity. Shown are three exemplary synapses with increasingly larger active vesicle pools, imaged in confocal (Syt1) and STED (TNR). The mean fluorescence intensities of TNR and Syt1, normalized to the medians of each respective experiment, are plotted against each other. The values for Syt1 were divided into five bins containing equal numbers of synapses. Quantification of the correlation between the intensities demonstrates a strong link between the size of the active vesicle pool and the amount of recycling TNR epitopes (*N* = 3 independent experiments, >1,100 synapses imaged per datapoint, Spearman’s ρ = 0.927, ***p < 0.001). **(B)** Newly-emerged TNRs were labeled in a similar fashion to panel a (magenta), and the neuronal membranes were visualized by incubation with the lipophilic dye DiO (green). Shown are exemplary images of postsynapses with increasingly larger head sizes. The mean fluorescence intensities of TNR and the mean synapse area, normalized to the medians of each respective experiment, are plotted against each other. The values for the synapse area were divided into five bins containing equal numbers of synapses. Quantification of the correlation between the intensities demonstrates a strong link between the size of the dendritic spine and the amount of recycling TNR epitopes (N = independent experiments, >280 synapses imaged per datapoint, Spearman’s ρ = 0.862, ***p < 0.001. Scale bars = 300 nm. The data shown in the plots represent the means (long horizontal lines) ± SEM (short horizontal lines), with individual dots indicating separate experiments. Adapted from Dankovich et al. (2021) with permission from Springer Nature (http://creativecommons.org/licenses/by/4.0/).

In further support of this mechanism, it was found that recycling TNR molecules are significantly enriched at synaptic regions, while more stable TNR molecules are present throughout the neuronal surface. In addition, it was demonstrated that TNR recycling is tightly linked to synaptic activity and strength: the amount of recycling TNR molecules detected at the neuronal surface increased following treatment with an activity-enhancing drug (the GABA_A_ channel blocker bicuculline) and decreased following treatment with activity-reducing drugs (the AMPA and NMDA channel blockers CNQX and AP5). The authors further established this link at the synapses themselves. To do so, they labeled actively recycling synaptic vesicles using antibodies against the lumenal domain of synaptotagmin1 (Syt1) as a proxy for local synaptic activity (Kraszewski et al., 1996; Wilhelm et al., 2010; Truckenbrodt et al., 2018; Gürth et al., 2020). Using stimulated emission depletion (STED) microscopy, they confirmed that local synaptic activity is significantly correlated to the extent of recycling. In a second experiment, the authors stained the neurons with lipophilic dye to reveal synaptic membranes, and found a second significant correlation between the extent of TNR recycling and the size of the postsynaptic head (which is known to be an important correlate of synaptic strength; Humeau and Choquet, 2019; Figure 4).

An interesting point to consider is the timespan of the TNR recycling loop (~3 days), which is considerably longer than that of other, well-studied recycling molecules (Bretscher, 1989; Koenig and Edwardson, 1997; Bridgewater et al., 2012). The authors provided a partial answer by metabolically labeling glycans with azide-carrying sugars and then visualizing these with fluorophores using a click chemistry reaction (Saka et al., 2014). This experiment revealed that recycling TNR molecules appear to become re-glycosylated throughout their intracellular trafficking route. This finding was further supported by immunostainings showing that intracellular recycling TNRs colocalize with somatic endoplasmic reticulum and Golgi apparatus following their internalization. Pathways of re-glycosylation have not been widely investigated, but there are several reports of this process occurring in non-neural cells (for example, in liver cells; Kreisel et al., 1988; Volz et al., 1995; Porwoll et al., 1998). While the biological function of this process remains to be established, one simple possibility is that it serves to repair the wear and tear of frequently recycling molecules without the need to replace their protein core. It is also possible that the glycans residues themselves play a role in the recycling process by functioning in the sorting of the proteins, as has been shown in non-neural cells (Scheiffele et al., 1995).

Besides their internalization for the purpose of re-glycosylation, it is also interesting to consider that ECM molecules may be internalized to activate intracellular signaling cascades. Recent findings have shown that several types of cell-surface receptors can undergo post-endocytic “internalized activation”, i.e., trigger distinct signaling activation from within intracellular compartments (Wang et al., 2021). It is, therefore, possible to imagine that internalized ECM-bound receptors may trigger signaling cascades related to, for example, synaptic plasticity.

The findings discussed in this review demonstrate that the neural ECM, while composed of extremely stable components, needs to remain far more freely modifiable than previously expected, due to the high rate of synapse changes in the living brain. The only solution proposed so far to this problem remains the possibility that the ECM molecules have an ability to be recycled. While this concept is novel in the context of synaptic plasticity, it has, in fact, already been reported in other cell types for the process of fibrillogenesis (Varadaraj et al., 2017). In the respective study, it was demonstrated that the ECM protein fibronectin could be internalized through the activity of integrin and TGF-β receptors, and then subsequently recycled re-integrated into extracellular fibrils. While this is the only demonstration, to our knowledge, of a complete recycling loop of an ECM molecule, many additional studies also add credence to the concept of ECM recycling at synapses. These include reports of ECM molecules that undergo internalization (e.g., Coopman et al., 1996; Tammi et al., 2001; Shi and Sottile, 2008; Lobert et al., 2010; Leonoudakis et al., 2014), demonstrations that ECM receptors are present at synapses (e.g., Kramár et al., 2002; Huang et al., 2006; Roszkowska et al., 2016; Izumi et al., 2017; Apóstolo et al., 2020; Briatore et al., 2020), and reports that synapses contain the machinery for trafficking recycling molecules in an activity-dependent manner (Tang, 2008; Gürth et al., 2020; Helm et al., 2021). Nevertheless, additional demonstrations of the recycling of ECM molecules in neurons are anticipated in the future. We expect such studies to rely on creative probes developed for studying molecular recycling *in vivo*, and on the current explosion in the development of high-resolution imaging methods, including tools that enable long-term imaging with limited phototoxicity (e.g., Bodén et al., 2021).

## Conclusion

Recycling mechanisms at synapses are well-studied for presynaptic vesicle release, where such a process is crucial for maintaining continuous neurotransmission without the need for a constant supply of vesicles. In a similar fashion, ECM recycling may also serve to preserve energy at the synapse by allowing continuous remodeling without the need for *de novo* synthesis and secretion of ECM components. While the energy gain is clear in the case of synaptic vesicles, it is not entirely obvious whether this also holds true for ECM recycling. Conceivably, this process also serves additional functions, for example, in cellular signaling. Considering that it appears to be largely synaptic and tightly linked to local activity, it is possible to imagine that this mechanism is intimately involved in synaptic function. In agreement with this claim, it was shown that perturbing TNR recycling with large antibody aggregates interfered severely with synaptic vesicle release and resulted in structural changes to the postsynapse (Dankovich et al., 2021). We predict that such perturbations to ECM recycling would also have implications for synaptic plasticity, both at the structural and the molecular level, and may also manifest in brain disorders. Hopefully, future studies will shed light on these ideas and reveal additional molecular details on the involvement of ECM recycling in synaptic function.

## Author Contributions

The manuscript was conceived by TD and SR, written by TD and revised by SR. All authors contributed to the article and approved the submitted version.

## Conflict of Interest

The authors declare that the research was conducted in the absence of any commercial or financial relationships that could be construed as a potential conflict of interest.

## Publisher’s Note

All claims expressed in this article are solely those of the authors and do not necessarily represent those of their affiliated organizations, or those of the publisher, the editors and the reviewers. Any product that may be evaluated in this article, or claim that may be made by its manufacturer, is not guaranteed or endorsed by the publisher.
